# Identification of an Immune-Related LncRNA Signature in Gastric Cancer to Predict Survival and Response to Immune Checkpoint Inhibitors

**DOI:** 10.3389/fcell.2021.739583

**Published:** 2021-10-13

**Authors:** Zuoyou Ding, Ran Li, Jun Han, Diya Sun, Lei Shen, Guohao Wu

**Affiliations:** ^1^Department of General Surgery, Zhongshan Hospital of Fudan University, Shanghai, China; ^2^State Key Laboratory of Medical Genomics, National Research Center for Translational Medicine at Shanghai, Shanghai Institute of Hematology, Ruijin Hospital Affiliated to Shanghai Jiao Tong University School of Medicine, Shanghai, China

**Keywords:** gastric cancer, tumor mutational burden, immune-related lncRNAs, immune checkpoint inhibitors, immunophenoscore

## Abstract

Immune microenvironment in gastric cancer is closely associated with patient’s prognosis. Long non-coding RNAs (lncRNAs) are emerging as key regulators of immune responses. In this study, we aimed to construct a prognostic model based on immune-related lncRNAs (IRLs) to predict the overall survival and response to immune checkpoint inhibitors (ICIs) of gastric cancer (GC) patients. The IRL signature was constructed through a bioinformatics method, and its predictive capability was validated. A stratification analysis indicates that the IRL signature can distinguish different risk patients. A nomogram based on the IRL and other clinical variables efficiently predicted the overall survival of GC patients. The landscape of tumor microenvironment and mutation status partially explain this signature’s predictive capability. We found the level of cancer-associated fibroblasts, endothelial cells, M2 macrophages, and stroma cells was high in the high-risk group, while the number of CD8^+^ T cells and T follicular helper cells was high in the low-risk group. Immunophenoscore (IPS) is validated for ICI response, and the IRL signature low-risk group received higher IPS, representing a more immunogenic phenotype that was more inclined to respond to ICIs. In addition, we found RNF144A-AS1 was highly expressed in GC patients and promoted the proliferation, migration, and invasive capacity of GC cells. We concluded that the IRL signature represents a novel useful model for evaluating GC survival outcomes and could be implemented to optimize the selection of patients to receive ICI treatment.

## Introduction

Gastric cancer (GC) is the fifth most common malignant tumor and the fourth leading cause of cancer-related deaths worldwide. According to recent statistics, GC patients usually have a poor prognosis, with a 5-year survival rate of less than 25% and an average overall survival (OS) of 7–10 months after diagnosis (Bray et al., 2018). The early asymptomatic nature of the disease contributes to the poor prognosis of GC, leading to the late diagnosis of GC and a high risk of distant metastasis. Currently, radical surgical resection is still the most effective method to significantly prolong the survival time of GC patients (Yu et al., 2019). Despite the development of postoperative adjuvant chemotherapy and targeted drugs in the last decade, the prognosis remains extremely poor for advanced GC patients (Coutzac et al., 2019).

Recent breakthrough in immunotherapy, most prominently using immune checkpoint inhibitors (ICIs), has yielded impressive results in several solid tumors and emerged as a novel optional treatment strategy for advanced GC (Kono et al., 2020). Inhibition of programmed death-1 (PD-1)/programmed death-ligand 1 (PD-L1) with ICIs, such as nivolumab and pembrolizumab, has entered clinical trials for GC patients (Kang et al., 2017; Janjigian et al., 2018). A meta-analysis for clinical trials with ICI for advanced GC or esophago-gastric junction tumors indicated that ICI treatment could provide modest survival benefit for advanced GC patients (Chen et al., 2019). Although ICI treatment is a promising treatment strategy, only a subset of GC patients can receive a survival benefit. Hence, a practical assessment model is urgently needed to assess the prognosis of patients with GC and response to ICI treatment.

Previously, long non-coding RNAs (lncRNAs) were believed to have no coding function and were considered as transcriptional noise. In fact, lncRNAs play an essential role in gene regulation (Statello et al., 2021). Recent studies have identified that many lncRNAs are aberrantly expressed in multiple cancers and involved in immune-related gene expression and function, thus affecting the tumor immune microenvironment (Mathy and Chen, 2017; Botti et al., 2019; Pang et al., 2020). LncRNAs should be increasingly considered as novel prognostic markers and therapeutic targets for human cancer.

In this study, we aimed to develop a novel immune-related lncRNA signature to predict the OS and response to ICI of GC patients. We investigated the relationship of the immune-related lncRNA (IRL) signature to clinicopathological characteristics and prognosis in The Cancer Genome Atlas Stomach Adenocarcinoma (TCGA-STAD) cohort. In addition, immune cell infiltration, mutation status, and immunophenoscore (IPS) associated with this signature in GC were also thoroughly explored. This signature may be implemented to predict the OS of GC patients and contribute to more precise ICI treatment for GC in the next future.

## Materials and Methods

### Data Acquisition

The RNA sequencing data (FPKM value), clinical information, and mutation data of GC patients were downloaded from TCGA. Patients lacking survival information were excluded from further evaluation. IRLs were obtained from the ImmLnc database^1^ (Li et al., 2020). The clinical information of GC samples is detailed in Supplementary Table 1. The IPSs of patients with GC were obtained from The Cancer Immunome Atlas (TCIA).^2^

### Establishment of an Immune-Related lncRNA Prognosis Model

The “limma” R package was used to identify differentially expressed genes (DEGs) between GC tissues and matched adjacent non-cancerous tissues. The significance criteria was set as | logFC | > 1 and *P*-value < 0.05. After integrating IRLs from the ImmLnc database, DELs were identified. From the perspective of clinical features of patients, we used the R package “caret” to randomly divide the TCGA-STAD cohort into training and test groups to make sure the consistency of the patient composition between training and test groups. In the training cohort, identified DELs were subjected to univariate Cox regression analysis using the “survival” R package to pinpoint potential IRLs of prognostic value. LASSO regression analysis was used to minimize the risk of overfitting, and multiple stepwise Cox regression method was applied to identify hub IRLs for constructing the prognostic model. The risk score was calculated using the following equation: β1 × gene1 expression + β2 × gene2 expression + … + β*n* × gene *n* expression, where β was the correlation coefficient generated by the multiple Cox regression analysis.

### Evaluation of the Established Immune-Related lncRNA Signature

According to the median risk score, GC patients were divided into high-risk and low-risk groups. The Kaplan–Meier analysis was conducted using “survminer” and “survival” R packages to evaluate the prognostic value of the IRL signature. The sensitivity and specificity of the IRL signature were evaluated in terms of the area under the curve (AUC) of receiver operating characteristic (ROC) using “survivalROC” R package. Risk score curve, survival scatter diagram, and heatmap were carried out using the “pheatmap” package. Univariate and multivariate Cox analyses using “survival” R package were conducted to demonstrate that the signature establishes an independent prognostic model. A prognostic nomogram was then constructed using “rms” R package to predict 1-, 2-, and 3-year OS of GC patients, and a concordance index (C-index) was calculated to determine the discrimination of the nomogram *via* a bootstrap method with 1,000 resamples. Calibration curves were performed to assess the accuracy of this nomogram. In addition, a decision curve analysis (DCA) was performed to evaluate the clinical usefulness. Gene set enrichment analysis (GSEA) performed by GSEA software (version 4.1.0, downloaded from http://www.gsea-msigdb.org/gsea/index.jsp) was applied to evaluate all genes based on their log_2_ fold changes and assess functions associated with different risk groups.

### Estimation of Tumor-Infiltrating Immune Cells in Different Risk Groups

To explore the association between tumor-infiltrating immune cells and the risk score, we used TIMER, CIBERSORT, CIBERSORT-ABS, QUANTISEQ, XCELL, MCP-counter, and EPIC algorithms to examine the status of immune infiltration among GC patients from TCGA database. Wilcoxon signed-rank test was applied to analyze the differences of tumor-infiltrating immune cell level between high-risk and low-risk groups. The relationship of risk score values and immune infiltrating cells was determined by Spearman correlation analysis. Boxplots for immune infiltrating cells in high-risk and low-risk groups were conducted using “ggpubr” R package. ESTIMATE algorithm was applied to explore the status of immune cell infiltration among two subgroups, in which R script was downloaded from https://sourceforge.net/projects/estimateproject/to calculate immune scores, stromal scores, and estimate scores.

### Mutation Analysis

Mutation data in the form of Mutation Annotation Format (MAF) was applied, and we used the “maftools” R package to analyze the mutation data.

### Immunophenoscore Analysis

The calculation process of immunophenoscore was detailed in a previous article (Charoentong et al., 2017). Briefly, according to a panel of immune-related genes yielded from random forest results, a sample-wise *Z* score was calculated. The IPS was calculated on an arbitrary 0–10 scale based on the sum of the weighted averaged *Z* score to predict the response of ICIs, where higher scores are associated with the increased response to ICIs. The IPS results of 20 solid cancers can be acquired in the site of https://tcia.at/home.

### Patients and Gastric Cancer Samples

GC tissue samples and matched adjacent normal gastric tissues were acquired from patients with gastric cancer who had undergone radical gastrectomy at the Department of Surgery, Zhongshan Hospital, Fudan University, from 2013 to 2014. This study was approved by the Ethics Committee of Zhongshan Hospital, Fudan University (Approval No. B2019-193R). Written informed consents were collected from all patients. No patient received preoperative chemotherapy. Clinicopathological variables were collected from all patients before surgery. All patients were followed up until December 2019. The eight lncRNA expressions were evaluated in eight pairs of GC tissues and matched adjacent non-cancerous tissues. The expression of RNF144A-AS1 was detected in 47 pairs of GC tissues and matched adjacent non-cancerous tissues. OS was calculated from the date of gastrectomy to the date of death or last follow-up. The clinical information of GC samples is detailed in Supplementary Table 2.

### Cell Culture

The human GC cell lines (HGC27, AGS, NCIN87, and SUN1) and human normal gastric epithelial cells (GES1) were obtained from the American Type Culture Collection (ATCC, United States) and cultured in RPMI 1640 medium (Gibco, United States) with 10% fetal bovine serum (Gibco, United States) supplemented with 1% penicillin and streptomycin (Invitrogen, United States) at 5% CO_2_ and 37°C.

### Quantitative Real-Time PCR

Total RNA from GC tissues and cell lines was extracted using TRIzol reagent (Invitrogen, United States). Quantitative real-time PCR (qRT-PCR) was performed as previously described (Li et al., 2019). The primers used for qRT-PCR are detailed in Supplementary Table 3.

### Western Blot

Western blot was performed as previously described (Li et al., 2019). The primary antibodies (N-cadherin, E-cadherin, vimentin, and β-actin) were purchased from Abcam, United States. The anti-mouse and anti-rabbit secondary antibodies were obtained from Cell Signaling Technology, United States.

### Transfection

Lentivirus packaging cells were transfected with LV3-pGLV-h1-GFP-puro vector (GenePharma, China) containing RNF144A-AS1 knockdown (sh-RNF144A-AS1-1 and sh-RNF144A-AS1-2) and a negative control sequence (NC), respectively. Lentiviral transduction was performed in HGC27 and AGS cells. Pools of stable transductants were generated by selection using puromycin (4 μg/ml) for 2 weeks.

### Colony-Forming Assay

The cells at the density of 5 × 10^2^ cells/well were seeded into a six-well plate and cultured for 2 weeks. The colonies were washed with PBS, fixed for 20 min with 100% methanol, and stained for 15 min with 1% crystal violet in 20% methanol. After washing three times with PBS, the number of colonies was calculated.

### Cell Counting Kit-8 Proliferation Assay

Cell Counting Kit-8 (CCK-8) proliferation assay (Dojindo, Japan) was used to detect cell viability. Cells were seeded in 96-well plates at a density of 3 × 10^3^ cells/well. After 10 μl CCK-8 reagent was added, cells were incubated at standard conditions for 1 h. Then, the absorbance at 450 nm was measured using a microplate reader (Bio-Rad Laboratories, United States).

### Wound-Healing Assay

The GC cells were seeded onto a coverslip at the density of 3 × 10^5^ cells/well in six-well plates. The monolayer was scratched with a sterile 20-μl pipette tip. The wound area was photographed under a light microscope (Leica, Wetzlar, Germany) at 0, 24, and 48 h.

### Transwell Cell Invasion Assay

Cell migration and invasion were measured using Transwell assay as described elsewhere (Lin et al., 2019).

### Statistical Analysis

The R software (version 4.0.2)^3^ was used to perform all statistical analyses. All data are represented in the format of mean ± SD from three independent experiments. Student *t*-test or one-way ANOVA was applied to evaluate differences between groups. *P* < 0.05 was considered as statistically significant.

## Results

### Construction of an Immune-Related lncRNA Signature Associated With the Prognosis of Gastric Cancer Patients

**FIGURE 1:**
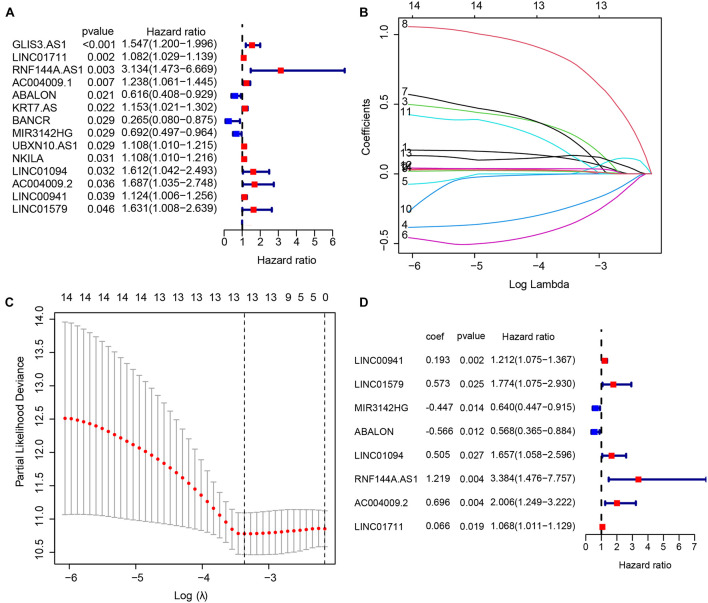
Construction of an immune-related long non-coding RNA (lncRNA) signature to predict the prognosis of patients with gastric cancer. **(A)** 14 immune-related lncRNAs associated with OS identified by the univariate Cox regression model. **(B,C)** LASSO regression was performed to identify the minimum criteria. **(D)** Coefficients of eight genes calculated by multivariate Cox regression.

In the TCGA-STAD cohort, 6,739 DEGs were identified between 375 GC samples and 32 adjacent normal samples. After integrating 3044 IRLs, we obtained 164 differentially expressed IRLs (Supplementary Table 4) including 30 down-regulated genes and 134 up-regulated lncRNAs. A univariate Cox regression analysis was performed among DELs to identify lncRNAs related significantly to OS, which produced 14 lncRNAs likely to carry a prognostic value (Figure 1A). LASSO regression was conducted to remove IRLs highly correlated with one another (Figures 1B,C). To further select key IRLs with greater prognostic value, multiple stepwise Cox regression was performed to obtain eight hub IRLs constructing an immune prognostic signature (Figure 1D).

### Evaluation of the Immune-Related lncRNA Signature in the TCGA Cohort

**FIGURE 2:**
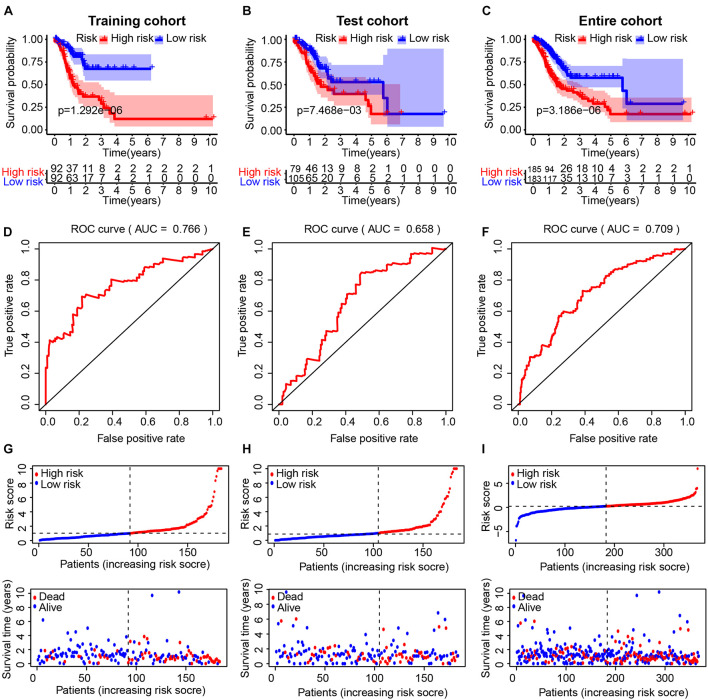
Evaluation of the immune-related lncRNA (IRL) signature in the TCGA cohort. The Kaplan–Meier curve analysis of the high- and low-risk groups in the training cohort **(A)**, test cohort **(B)**, and entire cohort **(C)**. Receiver operating characteristic (ROC) curve analysis of the IRL signature in the training cohort **(D)**, test cohort **(E)**, and entire cohort **(F)**. The distribution of risk scores and scatter plots of survival in patients in the training cohort **(G)**, test cohort **(H)**, and entire cohort **(I)**.

We divided GC patients into high- and low-risk groups according to the median risk score in the training, test, and entire cohort. A Kaplan–Meier analysis indicated that there was a significantly shorter OS in patients in the high-risk group (Figures 2A–C). The prognostic AUC value of this model achieved 0.766, 0.658, and 0.709 in the training, test, and entire cohort, respectively (Figures 2D–F). A higher risk score was associated with a higher likelihood that a patient would experience poor survival (Figures 2G–I).

### Stratification Analysis in the Cancer Genome Atlas Cohort

**FIGURE 3:**
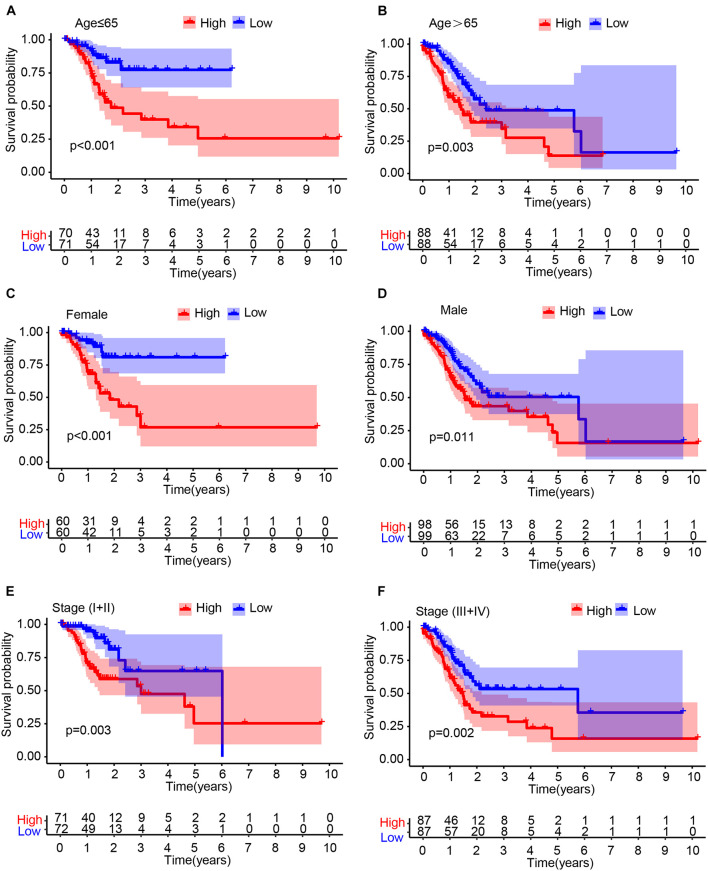
Stratification analysis. The Kaplan–Meier survival analysis of gastric cancer (GC) patients stratified by age **(A,B)**, male **(C,D)**, and stage **(E,F)**.

To determine whether the IRL signature was able to predict the prognosis of GC patient subgroups, we performed the Kaplan–Meier survival analysis in patients stratified by age (Figures 3A,B), gender (Figures 3C,D), and stage (Figures 3E,F), respectively. The results demonstrated that the IRL signature was able to distinguish low-risk from high-risk patients in different stratified groups.

### Construction of a Nomogram Model Integrated With the Immune-Related lncRNA Signature

**FIGURE 4:**
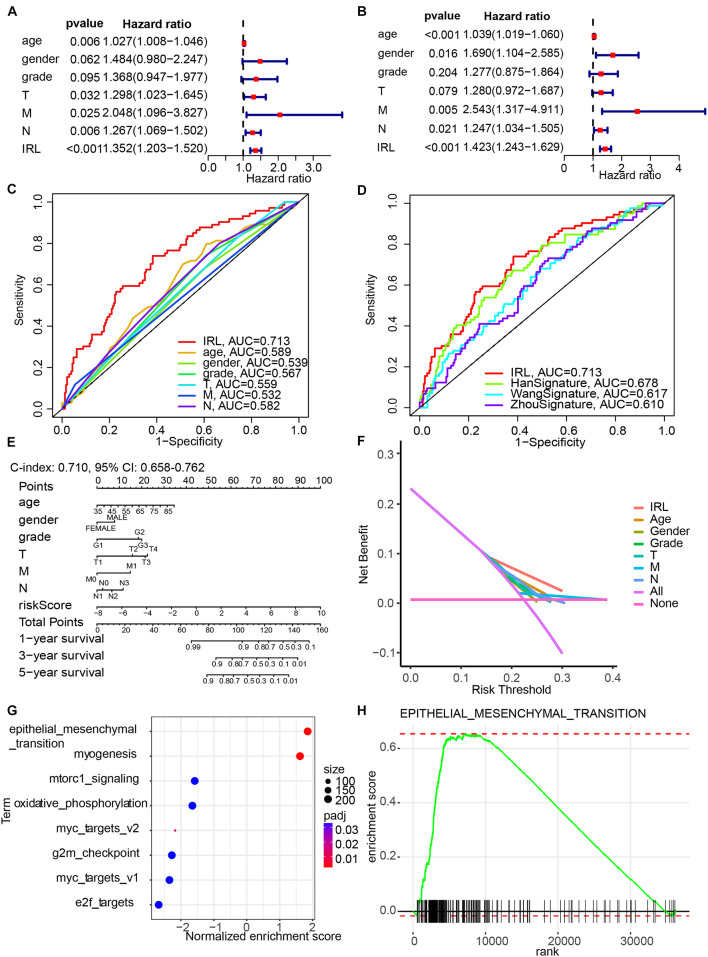
Construction of a nomogram model integrated with the IRL signature. **(A,B)** Univariate and multivariate Cox analyses included different clinicopathologic features. **(C,D)** Multivariable ROC curves for overall survival (OS). **(E)** Nomogram model for predicting the 1-, 3-, and 5-year OS of GC patients. **(F)** Decision curve analysis of the OS-related nomogram. “None” indicates that all samples were negative without intervention and the net benefit was 0. “All” indicates that all samples were positive with intervention. **(G)** The bubble plot of gene set enrichment analysis (GSEA) results. **(H)** The enrichment plot of epithelial–mesenchymal transition.

Univariate (Figure 4A) and multivariate (Figure 4B) Cox regression analyses indicated that IRL signature was able to independently predict the prognosis of GC patients. Multivariable ROC analysis demonstrated that the IRL signature possessed the highest prognostic accuracy (AUC = 0.713) compared to other factors, including age, gender, grade, T, M, and N (Figure 4C). Compared with the existing model, including the HanSignature (Han et al., 2021), WangSignature (Wang et al., 2021), and ZhouSignature (Zhou et al., 2021), our model showed a better prediction accuracy (Figure 4D). Subsequently, we constructed a nomogram-integrated IRL signature with other conventional prognosis factors and calculated its *C*-index (Figure 4E). The results of calibration curves demonstrated that the nomogram was able to accurately predict the OS of GC patients (Supplementary Figure 1). DCA of the nomogram was performed and revealed that the nomogram model had an excellent net benefit for GC patients’ OS (Figure 4F). In order to figure out which factors drive the different OS between high- and low-risk patients, we performed GSEA. The results indicated the epithelial–mesenchymal transition plays an important role in GC progression (Figures 4G,H).

### Investigation of Tumor-Infiltrating Immune Cells

**FIGURE 5:**
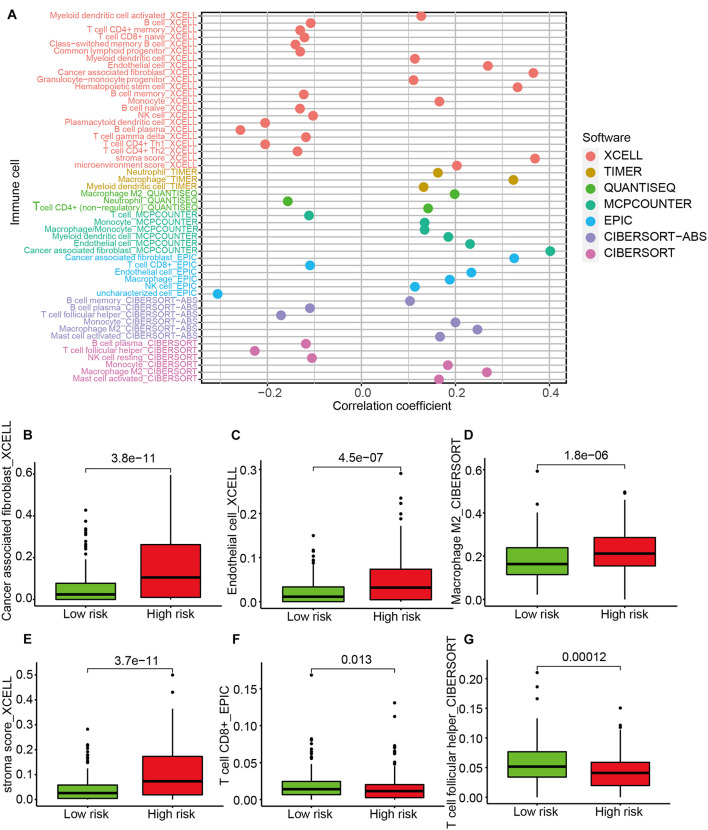
The analysis of tumor-infiltrating immune cells between the high-risk and low-risk groups. **(A)** Spearman correlation analysis. The type terms represent different algorithms. The abscissa indicates the correlation between risk score and immune cell infiltration. If the correlation > 0, the specific immune cell infiltration is positively associated with the risk score. If the correlation < 0, the specific immune cell infiltration is negatively associated with the risk score. The distribution levels of cancer-associated fibroblast **(B)**, endothelial cell **(C)**, macrophage M2 **(D)**, stroma score **(E)**, CD8^+^ T cell **(F)**, and T cell follicular helper **(G)** between the high-risk and low-risk groups.

A Spearman correlation analysis showed that the immune infiltrating status was significantly associated with risk score (Figure 5A). Specifically, the level of cancer-associated fibroblasts (Figure 5B), endothelial cells (Figure 5C), M2 macrophages (Figure 5D), and stroma score (Figure 5E) was higher in the high-risk group, while the expression of CD8^+^ T cells (Figure 5F) and T follicular helper (Tfh) cells (Figure 5G) was higher in the low-risk group. The results of ESTIMATE algorithm showed the immune score had no significant difference between the high- and the low-risk groups (Supplementary Figure 2A), while the stromal score was higher in the high-risk group (Supplementary Figure 2B). The results of correlation analyses between immune cell infiltration and risk score indicated that the terms of macrophages M2, monocytes, and mast cell resting were positively related to the risk score, while the terms of memory CD4 T cell activation, plasma cells, and T follicular helper cells were negatively related to the risk score (Supplementary Figure 2C).

### Tumor Mutational Burden Status Among Risk Groups

**FIGURE 6:**
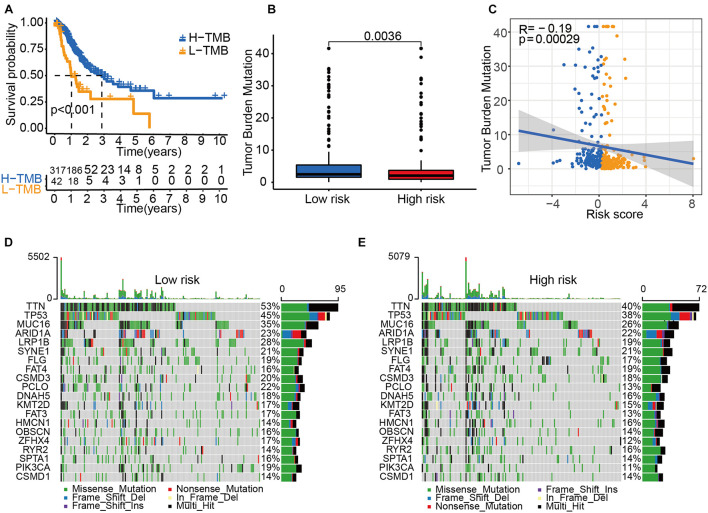
Tumor mutational burden (TMB) status among risk groups. **(A)** The Kaplan–Meier curve analysis of high- and low-TMB groups. **(B)** TMB in high- and low-risk groups. **(C)** A correlation analysis between TMB and risk score. Yellow dots represent the high-risk group. Blue dots represent the low-risk group. Mutation profile of the low-risk group **(D)** and the high-risk group **(E)**.

Next, we explored the role of tumor mutational burden (TMB) in the prognosis of GC patients. GC patients with higher TMB had a better OS (Figure 6A), and patients allocated to the low-risk group had a higher TMB than those allocated to the high-risk group (Figure 6B). The TMB was negatively associated with the risk score (Figure 6C). The mutation profile is illustrated in Figures 6D,E. We found that the mutation rate of most genes was high in the low-risk group except for SYNE1, FAT4, HMCN1, RYR2, and CSMD1. These genes with a high mutation rate could be potential biomarkers for predicting GC patients’ OS or responses to ICIs.

### The Association Between Risk Groups and Response to Immune Checkpoint Inhibitor

**FIGURE 7:**
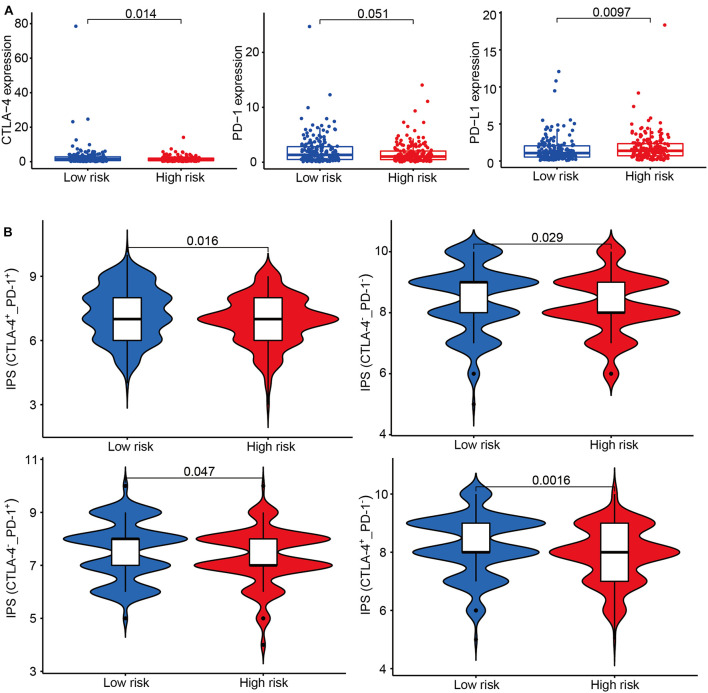
The association between risk groups and response to immune checkpoint inhibitors (ICI). **(A)** The gene expression of CTLA-4, PD-1, and PD-L1 in the high-risk and low-risk groups. **(B)** The association between IPS and the IRL signature of GC patients. Patients were divided into four groups: CTLA-4^+^_*PD*1^+^, CTLA-4^–^_*PD*1^–^, CTLA-4^+^_*PD*1^–^, and CTLA-4^–^_*PD*1^+^.

According to the transcriptional data, we found immune checkpoint-related genes were differently expressed between the high-risk and low-risk groups (Figure 7A). IPS has been confirmed to have a predictive value in melanoma patients treated with the CTLA-4 and PD-1 blockers (Van Allen et al., 2015; Charoentong et al., 2017). We used the immunophenoscore to evaluate whether the risk score could predict the response to ICIs in GC patients. The IPS was significantly higher in the low-risk group, which indicated the low-risk group patients had a better opportunity for ICI application (Figure 7B).

### RNF144A-AS1 Is Highly Expressed in Gastric Cancer and Promotes Cell Proliferation, Migratory, and Invasive Potential

**FIGURE 8:**
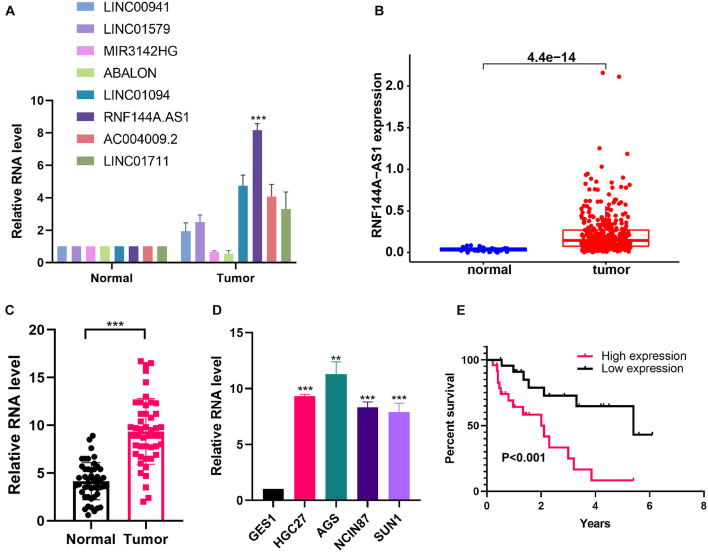
RNF144A-AS1 is highly expressed in GC tissues and cells. **(A)** The RNA expression of eight lncRNAs included in the IRL in normal and tumor tissues. **(B)** RNF144A-AS1 expression in The Cancer Genome Atlas Stomach Adenocarcinoma (TCGA-STAD) cohort. **(C)** RNF144A-AS1 expression in 47 pairs of GC tissues and marched adjacent non-cancerous tissues. **(D)** The expression of RNF144A-AS1 in human normal gastric epithelial cells and GC cell lines. **(E)** The Kaplan–Meier curve analysis of 47 GC patients. The patients were divided into high-risk and low-risk groups according to the median expression of RNF144A-AS1. All measurements are shown as the means ± SD from three independent experiments, **p < 0.01; ***p < 0.001.

To identify the key lncRNA involved in GC progression, we determined the expression of eight lncRNAs in the IRL signature. From the TCGA-STAD cohort, the eight lncRNAs were highly expressed in GC tissues except for LINC01579 (Supplementary Figure 3). We found that the difference in RNF144A-AS1 expression is the most significant between normal and tumor tissues (Figure 8A). We also found that RNF144A-AS1 was highly expressed in GC tissues from TCGA database (Figure 8B). Next, we analyzed RNF144A-AS1 expression in 47 pairs of GC tissues and matched adjacent non-cancerous tissues. The results indicated RNF144A-AS1 expression was upregulated in GC tissues (Figure 8C). Meanwhile, a higher expression of RNF144A-AS1 was observed in GC cells compared with human normal gastric epithelial cells (Figure 8D). Patients with a high expression of RNF144A-AS1 predicted poor overall survival (Figure 8E).

**FIGURE 9:**
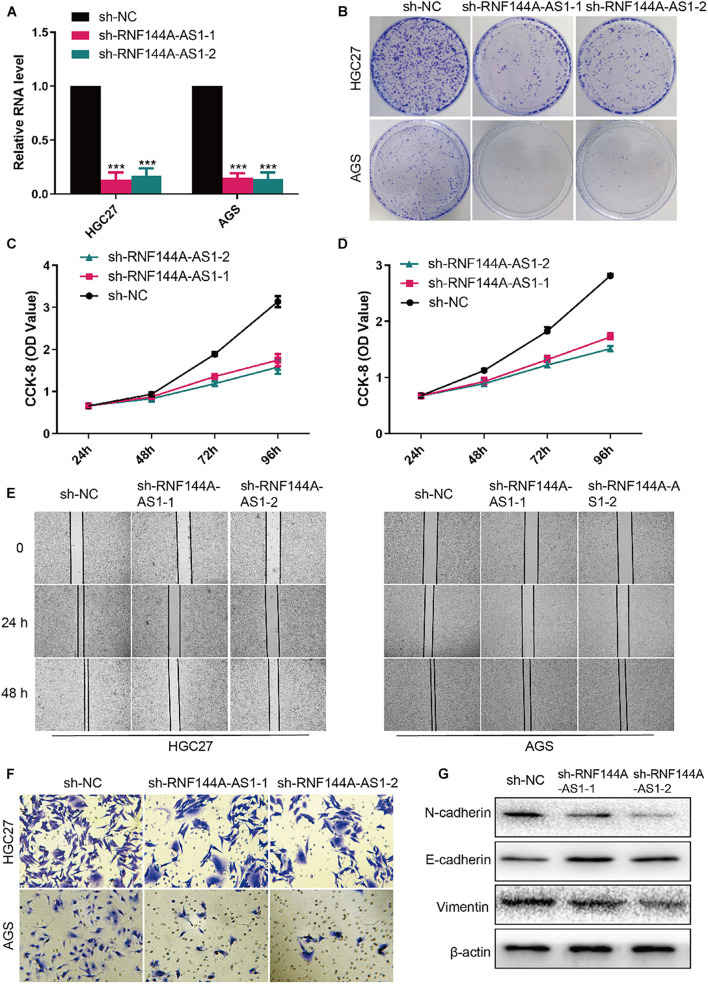
RNF144A-AS1 promotes cell proliferation, migratory, and invasive potential. **(A)** The efficiency of RNF144A-AS1 knockdown was detected by quantitative real-time PCR (qRT-PCR). **(B)** Representative pictures of colony-forming assay. A growth curve analysis showing the cell growth of HGC27 **(C)** and AGS **(D)** cells with RNF144A-AS1 knockdown. **(E)** The migration distance was measured to analyze the migration ability of GC cells that were treated with sh-NC or sh-RNF144A-AS1-1 or sh-RNF144A-AS1-2. **(F)** Transwell Matrigel invasion assay in GC cells with RNF144A-AS1 knockdown. **(G)** Representative WB images showing the protein level of N-cadherin, E-cadherin, and vimentin in GC cells with RNF144A-AS1 knockdown. All measurements are shown as the means ± SD from three independent experiments, ***p < 0.001.

RNF144A-AS1 expression was knocked down in HGC27 and AGS cells so that the biological role of RNF144A-AS1 in GC can be elucidated (Figure 9A). Colony-forming assay showed RNF144A-AS1 knockdown decreased the colony-forming ability in both HGC27 and AGS cells (Figure 9B). In CCK-8 proliferation assay, RNF144A-AS1 knockdown led to a significant proliferation reduction (Figures 9C,D). As shown in Figure 9E, RNF144A-AS1 knockdown decreased migration potential in both HGC27 and AGS cells. In Transwell invasion assay, a reduced number of invasive cells was observed in the RNF144A-AS1 knockdown group (Figure 9F). Combined with the GSEA results, we evaluated the expression of N-cadherin, E-cadherin, and vimentin to further explore the mechanisms of how RNF144A-AS1 affects GC cell phenotypes in HGC27 cells. The results revealed that RNF144A-AS1 knockdown decreased the expression of N-cadherin and vimentin, while increased E-cadherin expression (Figure 9G). Hence, the change of GC cell phenotypes induced by RNF144A-AS1 expression may be mediated by the activation of EMT signaling pathways.

## Discussion

Recent striking results from ICI treatment have provided a promising therapy option for GC patients. However, only a subset of patients could respond to the ICI therapy. It is urgent to identify predictive biomarkers for the response of ICI. It is unlikely to develop a single predictive biomarker because of the complexity of the tumor biology and immune response. The integration of multiple tumor and immune response parameters may contribute to accurate predictions (Masucci et al., 2016). Emerging evidence indicates that lncRNAs play a critical role in the immune system and the development of cancer by interacting with DNA, RNA, or proteins to regulate the expression of protein-coding genes (Atianand et al., 2017). It is of great significance to develop an IRL model to predict the OS and ICI response of GC patients.

Wang et al. (2021) developed an IRL prognostic signature according to the differentially expressed lncRNAs between high and low immune-infiltrating cell groups based on a single-sample gene-set enrichment analysis (ssGSEA) algorithm. We take another method to discover the potential DELs. Based on the hypothesis that if a lncRNA plays a critical role in the immune system, their correlated genes are supposed to be enriched in immune-related pathways, multiple lncRNA regulators that are associated with immune-related pathways were identified (Li et al., 2020). We acquired the IRLs of gastric cancer from this study and constructed a prognostic IRL signature. In addition, the published article only had a training set and lacked a validation set. We also explored the association between the IRL signature and tumor microenvironment (TME), mutation status, and IPS in gastric cancer. Moreover, we found RNF144A-AS1 deriving from our signature promotes GC through activating the EMT signaling pathway.

Eight IRLs are involved in the established signature. LINC00941 has been confirmed to exhibit pro-tumorigenic and pro-metastatic abilities during tumorigenesis. RNF144A-AS1 was found to be an oncogene in bladder cancer, which promotes proliferation, migration, and invasion in tumor progression by regulating SOX11 *via* sponging miR-455-5p (Bi et al., 2020). However, its role and function in gastric cancer has not been elucidated. LINC00941 is highly expressed in GC samples and promotes GC progression by affecting tumor depth and distant metastasis (Liu et al., 2019). LINC01579 was found to promote glioblastoma cell proliferation in a ceRNA manner of absorbing miR-139-5p to increase EIF4G2 expression (Chai and Xie, 2019). MIR3142HG is a critical regulator of the inflammatory response, and the attenuated expression of MIR3142HG/miR-146a contributes to the reduced inflammatory response in IPF fibroblast (Hadjicharalambous et al., 2018). As for LINC01094, several studies reported that it is a pro-tumorigenic lncRNA, and microRNA-184 (Xu H. et al., 2020), miR-126-5p (Li and Yu, 2020), miR-577 (Xu J. et al., 2020), miR-330-3p (Zhu et al., 2020), and miR-224-5p (Jiang et al., 2020) were identified as its targets. The role of ABALON, AC004009-2, and LINC01711 has not been explored by biological assays, providing directions and clues for future research. Moreover, all the published studies mentioned above focused on the role of IRLs in the proliferation, invasion, and migration of cancer cells but largely ignored its role in the immune system.

The TME is complex and constantly evolving. It consists of stromal cells, fibroblasts, endothelial cells, and innate and adaptive immune cells (Hinshaw and Shevde, 2019). The cross-talk of these cells determines the fate of tumor cells. A greater understanding of the TME will contribute to improving the prognosis of cancer patients. We applied seven different algorithms to examine the immune infiltration status between the high-risk and low-risk groups. High infiltration of M2 macrophages in cancerous tissues is considered as a negative prognosis in gastric cancer, breast cancer, lung cancer, hepatoma, and other malignancies (Cardoso et al., 2014; Wang et al., 2018). Other components of the TME, such as cancer-associated fibroblasts (CAFs), endothelial cells, and stromal cells, also play a role in the development of cancer (Hinshaw and Shevde, 2019). CAFs possess wound-healing ability and are found to promote tumor proliferation, invasion, and metastasis. Also, CAFs could secrete immune-suppressive cytokines that polarize macrophages to the M2 phenotype and lead to the exhaustion and depletion of CD8^+^ T cells (Lakins et al., 2018). Hence, it is not surprising that the level of CAFs, endothelial cells, M2 macrophages, and stroma cells was high in the high-risk group. The presence of Tfh cells has been positively associated with long-term survival of patients with breast cancer or colorectal cancer (Bindea et al., 2013). Tfh cells are found to produce CXCL13 that plays an immune-protective role in anti-tumor immunity (Crotty, 2019). Our results indicated the numbers of CD8^+^ T cells and Tfh cells are higher in the low-risk group. In addition, the term of NK cell from the EPIC algorithm is contrary to the term of NK cell resting from the CIBERSORT algorithm. The term of NK cell consists of NK cell resting and NK cell activation. Hence, the explanation of this contrast may be that the NK cell resting is negatively associated with a risk score, and when considering the whole level of NK cells, the relationship changes into a positive association. Taken together, our findings show that tumor–immune cross-talk and tumor–stromal cross-talk might play a role in the prognosis of patients with GC.

We demonstrate that the IRL signature low-risk group has higher TMB, which means higher TMB was related to a better prognosis in this study. This finding may partially explain the predictive value of this model. Also, the TMB-high group exhibited a better OS in the TCGA-STAD cohort, which was consistent with previous studies (Wang et al., 2019; Zhang et al., 2020). Recently, the question of whether TMB status could be considered as a general prognostic biomarker to predict patients’ OS has been proposed, and several studies indicated that high tumor mutation burden failed to predict immune checkpoint blockade response across all cancer types (Passaro et al., 2020; McGrail et al., 2021). In the future, more study should be performed to determine the predictive value of TMB in GC patients.

The association between IPS and our IRL signature in GC was explored. We found that the IRL signature low-risk group had a better chance to receive ICI treatment and may stand for an immunogenic tumor microenvironment. These results indicate that the IRL signature was a potential model to determine which GC patients are more inclined to respond to ICI.

In conclusion, we constructed an IRL-based prognostic model, which is a reliable and accurate model to predict the OS and ICI response of GC patients. This signature may be implemented to improve GC prognosis and, in the future, to inform treatment with novel immunotherapies.

## Data Availability Statement

The original contributions presented in the study are included in the article/Supplementary Material, further inquiries can be directed to the corresponding author/s.

## Ethics Statement

The studies involving human participants were reviewed and approved by the Ethics Committee of Zhongshan Hospital, Fudan University (Approval No. B2019-193R). The patients/participants provided their written informed consent to participate in this study.

## Author Contributions

ZD, RL, JH, and GW conceived, revised the manuscript, and designed the study. ZD, DS, and LS performed the experiments. ZD, RL, and JH conducted the statistical analysis. ZD wrote the manuscript. All authors contributed to the article and approved the submitted version.

## Conflict of Interest

The authors declare that the research was conducted in the absence of any commercial or financial relationships that could be construed as a potential conflict of interest.

## Publisher’s Note

All claims expressed in this article are solely those of the authors and do not necessarily represent those of their affiliated organizations, or those of the publisher, the editors and the reviewers. Any product that may be evaluated in this article, or claim that may be made by its manufacturer, is not guaranteed or endorsed by the publisher.

## Footnotes

1
http://bio-bigdata.hrbmu.edu.cn/ImmLnc/

2
https://tcia.at/home

3
https://www.r-project.org/

## Supplementary Material

Supplementary Figure 1 Calibration plots of the nomogram-predicted probability of 1-, 2-, and 3-year OS.

Supplementary Figure 2 **(A,B)** The results of the ESTIMATE algorithm in the high-risk and the low-risk groups. **(C)** The correlation analyses between CIBERSORT-Results and risk score.

Supplementary Figure 3 The expression of eight genes between tumor and normal tissues in the TCGA-STAD cohort.

Supplementary Table 1 Baseline characteristics of the patients in the TCGA cohorts.

Supplementary Table 2 Association of RNF144A-AS1 expression with clinicopathologic characteristics of gastric cancer patients.

Supplementary Table 3 The primers used for qRT-PCR.

Supplementary Table 4 One hundred sixty-four differentially expressed IRGs between GC patients and healthy individuals.
