# Supplementary figures and images for: Identification of an Immune-Related LncRNA Signature in Gastric Cancer to Predict Survival and Response to Immune Checkpoint Inhibitors
Source: Front Cell Dev Biol. 2021 Oct 13;9:739583. doi: 10.3389/fcell.2021.739583 (PMC8548421; doi:10.3389/fcell.2021.739583)

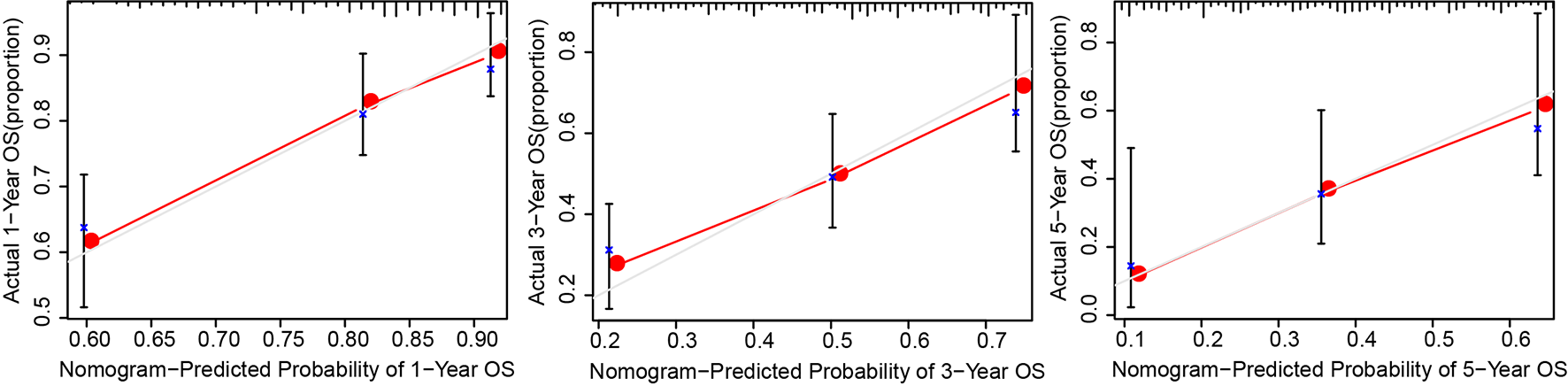

Supplement: Supplementary Figure 1 — Calibration plots of the nomogram-predicted probability of 1-, 2-, and 3-year OS. [file Image_1.TIF]

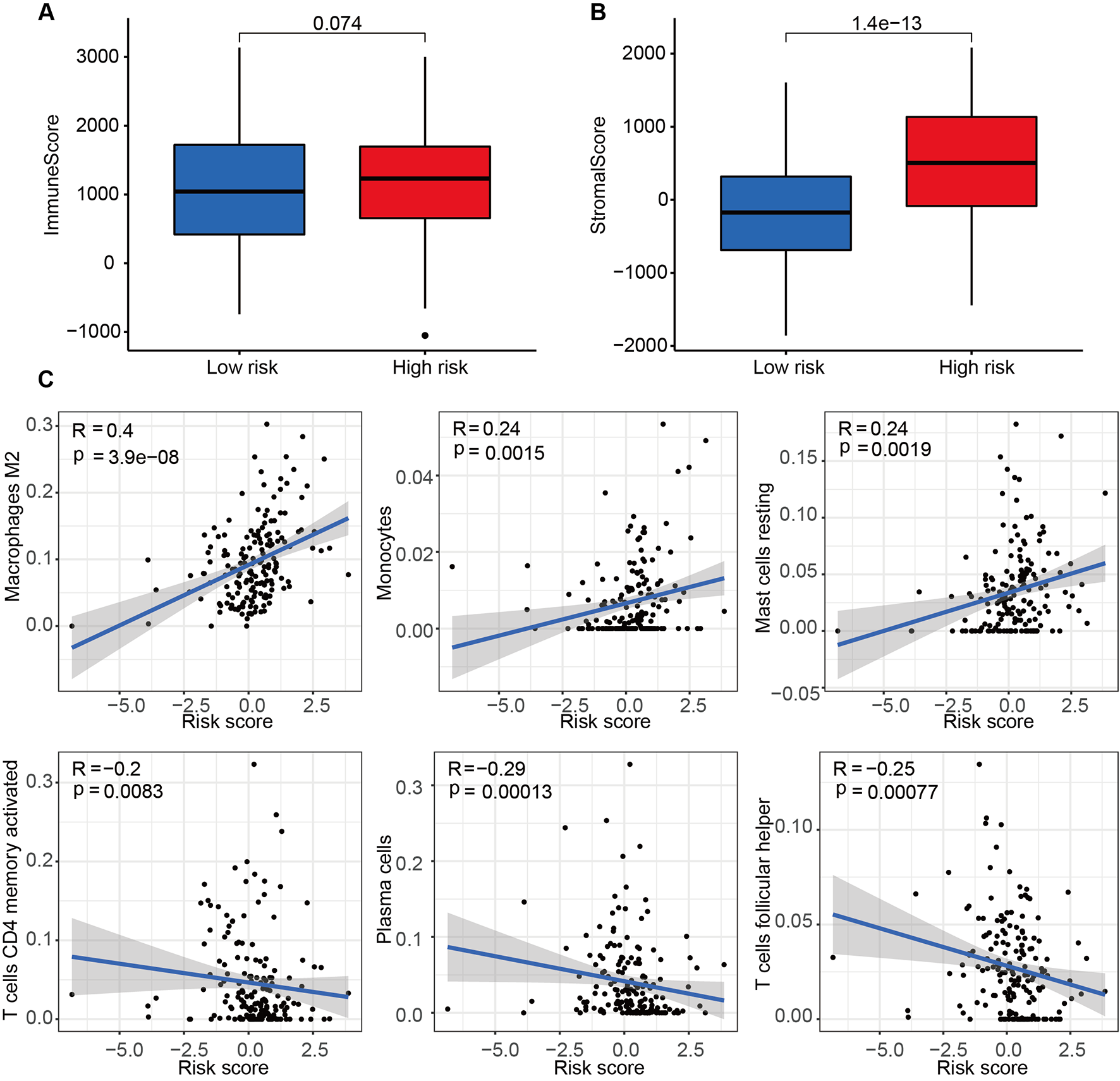

Supplement: Supplementary Figure 2 — (A,B) The results of the ESTIMATE algorithm in the high-risk and the low-risk groups. (C) The correlation analyses between CIBERSORT-Results and risk score. [file Image_2.TIF]

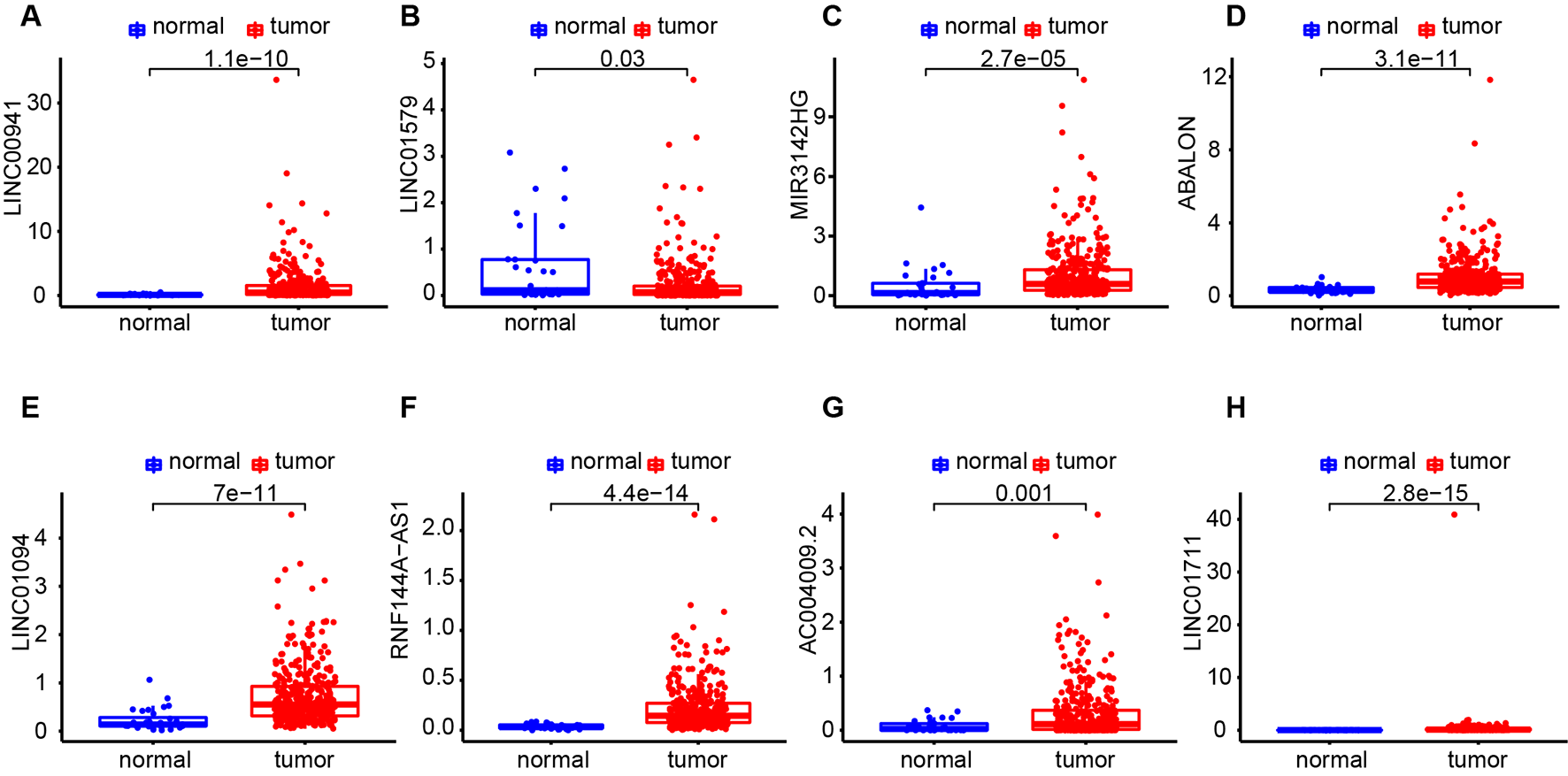

Supplement: Supplementary Figure 3 — The expression of eight genes between tumor and normal tissues in the TCGA-STAD cohort. [file Image_3.TIF]
